# Co_3_O_4_ Supported on Graphene-like Carbon by One-Step Calcination of Cobalt Phthalocyanine for Efficient Oxygen Reduction Reaction under Alkaline Medium

**DOI:** 10.3390/nano13071241

**Published:** 2023-03-31

**Authors:** Huang Tan, Xunyu Liu, Miaohui Wang, Hui Huang, Peipei Huang

**Affiliations:** 1 School of Physics and Information Technology, Shaanxi Normal University, No. 620, West Chang’an Avenue, Chang’an District, Xi’an 710119, China; 2 Jinduicheng Molybdenum Group Company Limited, Weinan 714102, China; 3 Institute of Functional Nano and Soft Materials Laboratory (FUNSOM), Jiangsu Key Laboratory for Carbon-Based Functional Materials & Devices, Soochow University, 199 Ren’ai Road, Suzhou 215123, China

**Keywords:** Co_3_O_4_, graphene-like carbon, oxygen reduction reaction, one-step calcination, cobalt phthalocyanine

## Abstract

Exploiting cost-effective and durable non-platinum electrocatalysts for oxygen reduction reaction (ORR) is of great significance for the development of abundant renewable energy conversion and storage technologies. Herein, a series of Co_3_O_4_ supported on graphene-like carbon (Co_3_O_4_/C) samples were firstly effectively synthesized by one-step calcination of cobalt phthalocyanine and their electrocatalytic performances were measured for ORR under an alkaline medium. By systematically adjusting the calcination temperature of cobalt phthalocyanine, we found that the material pyrolyzed at 750 °C (Co_3_O_4_/C−750) shows the best ORR electrocatalytic performance (half-wave potentials of 0.77 V (vs. RHE) in 0.1 M KOH) among all the control samples. Moreover, it displays better stability and superior methanol tolerance than commercial 20% Pt/C. The further electrochemical test results reveal that the process is close in characteristics to the four-electron ORR process on Co_3_O_4_/C−750. In addition, Co_3_O_4_/C−750 applied in the zinc–air battery presents 1.34 V of open circuit potential. Based on all the characterizations, the enhanced electrocatalytic performances of Co_3_O_4_/C−750 composite should be ascribed to the synergistic effect between Co_3_O_4_ and the graphene-like carbon layer structure produced by pyrolysis of cobalt phthalocyanine, as well as its high specific surface area.

## 1. Introduction

The oxygen reduction reaction (ORR) is of great significance for the development of abundant renewable energy conversion and storage technologies with low environmental pollution, such as fuel cells (FCs) and metal–air batteries [1,2,3,4,5]. However, the inherent sluggish kinetics of ORR hinders the extensive application of FCs and metal–air batteries. Platinum and platinum-based electrocatalysts have been proven to enable the high catalytic activity in ORR. However, the high cost and sensitivity to poisoning of Pt-based electrocatalysts greatly impede their commercial applications [6,7,8]. Therefore, exploring cost-effective and durable non-platinum catalysts for ORR has been drawing more and more attention.

Nonprecious transition metal-based oxides (perovskite [9], spinels and pyrochlore [10,11,12,13], single oxide [14,15], and multiple oxides [16], etc.), sulfides [17], nitrides [18], phosphides [19], organometallic compounds [20], and carbon-based materials [21,22] have been extensively studied for ORR. Among them, Co_3_O_4_ with mixed valences of Co cations (i.e., Co^2+^ and Co^3+^) seems to be one of the most competitive candidates for use in ORR due to their advantages of low cost and abundant reserves. The Co^2+^ ions are located on 1/8 of the tetrahedral ‘A’ sites and the Co^3+^ ions occupy 1/2 of the octahedral ‘B’ sites [23,24,25]. The presence of mixed valences of Co cations in the crystal structure could provide donor–acceptor chemisorption sites for the reversible adsorption of oxygen, thus favouring the ORR process [10,26,27]. Moreover, integrating the carbon-based materials with Co_3_O_4_ can further improve the relatively high electrocatalytic activity and enhance the durability generated by a synergistic effect between the two components [28,29,30,31,32,33]. However, there are only several works which report on the combination of Co_3_O_4_ and carbon-based materials via a one-step synthesis method for ORR.

In this work, a series of Co_3_O_4_ supported on graphene-like carbon (Co_3_O_4_/C) samples were firstly effectively synthesized by one-step calcination of cobalt phthalocyanine and their electrocatalytic performances for ORR was measured under alkaline medium conditions. By systematically adjusting the calcination temperature of CoPc, the sample pyrolyzed at 750 °C (Co_3_O_4_/C−750) was shown to have the best ORR electrocatalytic performance, with half-wave potentials of 0.77 V (vs. RHE) in 0.1 M KOH and an approximate four-electron ORR process. The stability and methanol tolerance of Co_3_O_4_/C−750 are better than those of commercial 20% Pt/C. In addition, Co_3_O_4_/C−750 applied in the zinc–air battery presents 1.34 V of open-circuit potential. Based on all the characterizations, the enhanced electrocatalytic performances of Co_3_O_4_/C−750 composite should be attributed to the synergistic effect between Co_3_O_4_ and the graphene-like carbon layer structure produced by pyrolysis of CoPc, as well as its high-specificity surface area.

## 2. Experimental Section

### 2.1. Materials

Except for the specific statement, all chemicals and reagents were purchased from China National Medicines Corporation (Shanghai, China), which were all analytical reagents and used without any further purification.

### 2.2. Preparation of Co_3_O_4_/C

In a typical procedure, 5 mL 0.1 mol·L^−1^ CoPc was dispersed in 50 mL water and stirred constantly. After that, 3 g NaCl and 3 g KCl were added into the above solution to form a homogeneous solution. Then, the sample solution was freeze-dried. Subsequently, the freeze-dried sample powder was calcined under different temperatures (700 °C, 750 °C, 800 °C, 900 °C and 1000 °C) in N_2_ for 2 h, with a temperature increase rate of 5 °C·min^−1^. The final product was repetitively washed with water and anhydrous ethanol and dried at 80 °C overnight. The different samples, pyrolyzed from 700 to 1000 °C, were named Co_3_O_4_/C−700, Co_3_O_4_/C−750, Co_3_O_4_/C−800, Co_3_O_4_/C−900 and Co_3_O_4_/C−1000, respectively.

### 2.3. Characterization

Thermogravimetric analysis (TGA) curve was recorded using a Pyris-Diamond TG/DTA instrument (Perkin Elmer, Waltham, MA, USA) in flowing N_2_ at a heating rate of 10 °C·min^−1^. X−ray diffraction patterns of the resultant products were characterized using an Empyrean, Holland Panalytical X−ray diffractometer (PANalytical B.V., Almelo, Holland) with Cu Kα radiation (λ = 0.154178 nm). Transmission electron microscope (TEM) and high−resolution TEM (HRTEM) images were obtained with a FEI−Tecnai F20 (200 kV, FEI, Hillsboro, America). A scanning electron microscope (SEM, Zeiss, Oberkochen, Germany) was used in the characterization of the surface morphology. An energy−dispersive spectrometer (EDS) was used to check the element contents of the samples. X−ray photoelectron spectroscopy (XPS) measurements were conducted on a KRATOS Axis ultra-DLD X−ray photo-electron spectroscope (Kratos Analytical Ltd, Manchester, UK) with a monochromatic Al Kα X−ray source. The Brunauer–Emmett–Teller (BET, Micromeritics, Atlanta, GA, USA) specific surface area of the samples was recorded using an ASAP 2050 medium with a high-pressure physical adsorption apparatus.

### 2.4. Electrochemical Measurements

Electrochemical correlation characterizations were performed by using a CHI 920C electrochemical workstation (CH Instruments, Chenhua, Shanghai, China) to explore the electrochemical properties of ORR in a standard three-electrode electrochemical cell. All tests were carried out at room temperature (25 °C) with a RRDE-3A Rotating Ring Disk Electrode Apparatus (ALS Co., Ltd., Tokyo, Japan). Graphite and Ag/AgCl were used as the counter electrode and the reference electrode, respectively. The rotating disk electrode (3 mm diameter, RDE) and rotating ring disk electrode (4 mm diameter, RRDE) were used as working electrodes. In a typical process for the preparation of working electrode, 5 mg sample was dispersed in 1 mL 0.5 wt.% Nafion aqueous solution. Then, the mixed solution was subjected to ultrasonic treatment for 1 h to acquire a uniform solution (~5 mg·mL^−1^). Subsequently, for RDE, 4 μL uniform catalyst solution was dropped onto the glassy carbon electrode surface. The catalyst loading amount was calculated to be 0.28 mg·cm^−2^, while 7.1 μL uniform catalyst solution was dropped onto the RRDE to achieve the identical loading amount. Finally, these prepared working electrodes were dried naturally in the air. The Pt/C (20 wt%, Johnson Matthey (Shanghai, China) Catalyst Co., Ltd., Shanghai, China) electrode was prepared with the same procedure. All experiments were conducted in 0.1 M KOH. The electrolyte was saturated with N_2_ (or O_2_) for at least 30 min before each test and the gas flow was maintained during the experiments. The cyclic voltammetry (CV) tests of catalysts were performed in O_2_-saturated and N_2_-saturated 0.1 M KOH, respectively. The linear sweep voltammogram (LSV) measurements were obtained in an O_2_-saturated electrolyte solution under 1600 revolutions per minute (rpm). All test obtained potentials were converted into the standard reversible hydrogen electrode (RHE) scale in accordance with the formula E_(RHE)_ = E_(Ag/AgCl)_ + 0.059 × pH + 0.199 V. Similarly, the stability tests were carried out in O_2_-saturated 0.1 M KOH under 1600 rpm.

## 3. Results and Discussion

### 3.1. Morphological and Structural Characterization

As is common knowledge, the CoPc has been known to exhibit electrocatalytic activity since it was proven in 1964 [34]. Inspired by this, the CoPc was chosen as the raw material to use to obtain the Co_3_O_4_. During the procedure of sample preparation, we found that the addition of NaCl and KCl could promote the dissolution of CoPc in water and that the above two salts could be removed thoroughly from the final product system via water rinse. Figure S1a shows the structure diagram of CoPc, which is a well-known metal–ligand coordination organic molecule whose derivatives are usually applied in oxygen reduction reactions [30]. In the following experiments, the thermogravimetric analysis (TGA) of CoPc was performed under N_2_ and the results are shown in Figure S1b. As can be seen, the mass of CoPc begins to decrease significantly at about 600 °C. When the temperature surpasses 700 °C, the quality of CoPc drops sharply, indicating that its structure begins to collapse at this temperature. Therefore, the calcination temperature was chosen from 700 °C. As the temperature goes up, CoPc could gradually transform into Co_3_O_4_ and carbon, details of which will be discussed in the following section.

**Figure 1:**
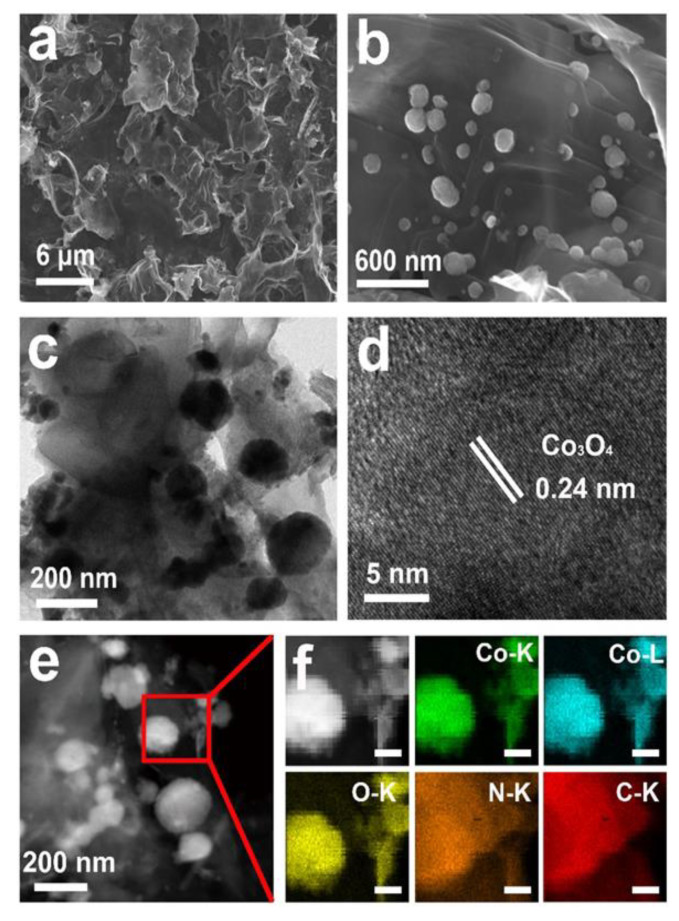
(**a**) SEM image, (**b**) Enlarged SEM image, (**c**) TEM image and (**d**) HRTEM image of Co_3_O_4_/C−750. (**e**) HAADF-STEM image and (**f**) relevant element analysis diagram of Co_3_O_4_/C−750 (scale bar for illustration: 50 nm).

There is abundant stacked graphene-like carbon in Co_3_O_4_/C−750, as shown in Figure 1a. The presence of Co_3_O_4_ nanoparticles with a diameter of around 180 nm separately and their evenly deposited nature on the surface of graphene-like carbon layers are confirmed in the magnified SEM image (Figure 1b). As presented in Figure S2, the SEM images of Co_3_O_4_/C−700, Co_3_O_4_/C−800, Co_3_O_4_/C−900 and Co_3_O_4_/C−1000 demonstrate that as the temperature increases, the morphology of graphene-like carbon layers gradually changes from thin flake to thick bulk. The average sizes of separate Co_3_O_4_ particles in the SEM images are around 80 nm; however, at a higher calcination temperature, more CoPc decompose and transform into Co_3_O_4_ and carbon. Obviously, more Co_3_O_4_ aggregates appear on the carbon support instead of separate particles. Therefore, the calcination temperature has a great influence on both the morphology of the resultant carbon and the dispersion of Co_3_O_4_, which can further affect the catalytic properties of the composites.

The TEM image of Co_3_O_4_/C−750 (Figure 1c) shows that the sample has a lamellar structure, which is similar to that of graphene. Clearly, separate Co_3_O_4_ nanoparticles with sizes pf around 100~200 nm can be observed on the graphene-like carbon layer. The high-resolution TEM (HRTEM) image of Co_3_O_4_/C−750 is displayed in Figure 1d, where the lattice spacing of 0.24 nm is corresponds well with the Co_3_O_4_ (311) plane. The HAADF-STEM and relevant element characterization were performed to further reveal the constituent of Co_3_O_4_/C−750. Corresponding to the STEM pattern of Co_3_O_4_/C−750 (Figure 1e), Figure 1f shows that the Co (green and blue), O (yellow), N (orange) and C (red) elements are evenly distributed in the composite.

**Figure 2:**
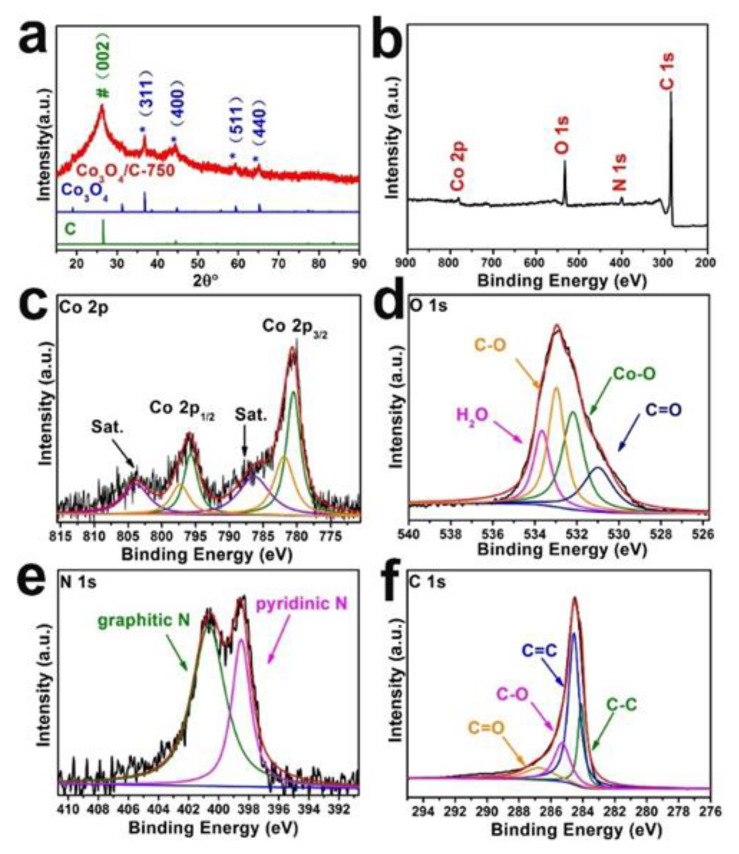
(**a**) XRD patterns of Co_3_O_4_/C−750, Co_3_O_4_, and graphite (red, blue and green traces, respectively, peaks labeled with * are attributed to Co_3_O_4_ and peak labeled with # is ascribed to C). XPS characterizations of Co_3_O_4_/C−750 for (**b**) survey spectrum, high-resolution analysis of (**c**) Co 2p, (**d**) O 1s, (**e**) N 1s and (**f**) C 1s.

X−ray diffraction (XRD) measurements were carried out to explore the crystal phase of Co_3_O_4_/C-750. As shown in Figure 2a, the characteristic peaks located at 36.9°, 44.8°, 59.4° and 65.2° are well indexed to (311), (400), (511) and (440) planes of Co_3_O_4_ (JCPDS No. 43–1003), respectively. The strongest intensity of the peak appearing at about 26° can be assigned to the (002) facet of graphite carbon (JCPDS No. 65–6212) in the XRD patterns. Except for the mentioned peaks, there is no other signal. The positions and relative intensities of these peaks are consistent with those on the standard cards, demonstrating that the sample is indeed composed of Co_3_O_4_ and graphitized carbon. The conclusion agrees with the TEM result. At the same time, the XRD measurements were performed on other samples obtained from different calcination temperatures shown in Figure S3. As shown in illustration, there are also several characteristic peaks located at 36.9°, 44.8°, 59.4°and 65.2°, which corresponding to the (311), (400), (511) and (440) planes of Co_3_O_4_ (JCPDS No. 43–1003). Notably, as the temperature increases, relative to the enhanced crystallinity of Co_3_O_4_, the graphitization of carbon significantly improves, which is accordance with the SEM results.

**Figure 3:**
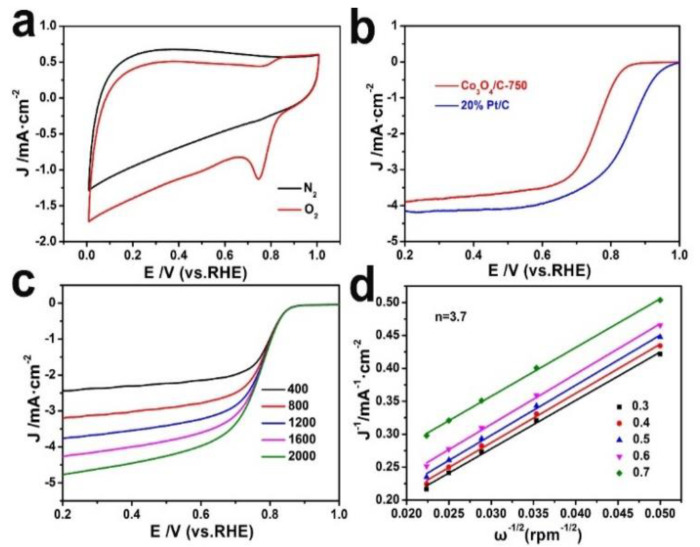
(**a**) CV curves of Co_3_O_4_/C−750 in N_2_− (black trace) and O_2_− (red trace) saturated 0.1 M KOH solution at a sweeping rate of 50 mV·s^−1^, respectively. (**b**) LSV curves of Co_3_O_4_/C−750 and 20% Pt/C in O_2_−saturated 0.1 M KOH at 1600 rpm with 10 mV·s^−1^ sweep rate. (**c**) LSV curves for Co_3_O_4_/C−750 at different rotation speeds (400–2000 rpm). (**d**) K−L plots of Co_3_O_4_/C−750 at different electrode potentials (V vs. RHE).

To explore the detailed information about the elemental character and oxidation state of catalysts, X-ray photoelectron spectroscopy (XPS) was employed. As shown in the XPS survey spectrum of Co_3_O_4_/C−750 (figure performance increases), this technique manifests the existence of Co, C, N and O elements. No signal of Na, K or Cl elements was detected, evidencing that NaCl and KCl were indeed all removed during the water washing procedure. With regard to the high-resolution XPS spectrum of Co 2p (Figure 3c), it can be deconvoluted with three peaks, corresponding to Co^3+^ (780.4eV and 795.5 eV), Co^2+^ (781.6eV and 797.3 eV) and the satellite peaks (786.3 eV and 803.8 eV) [35,36]. Similarly, the high-resolution XPS spectrum of O 1s (Figure 2d) is clearly identified by the C=O (531.0 eV), Co–O (532.2 eV), C–O (532.9 eV) and surface adsorbed water (533.6 eV) [37,38,39]. Meanwhile, two single N species peaks in the spectrum XPS of N 1s (Figure 2e) correspond to graphitic N (400.6 eV) and pyridinic N (398.5 eV), respectively [40]. Figure 2f shows four peaks corresponding to C–O (284.1 eV), C=C (284.5 eV), C–O (285.2 eV) and C=O (286.7 eV) [37,41,42,43,44]. The above results demonstrate that Co_3_O_4_/C−750 was successfully synthesized.

Because of the special morphology of Co_3_O_4_/C, the specific surface areas of samples prepared with different calcination temperatures were measured. Figure S4 shows the N_2_ adsorption–desorption isotherms of samples prepared at different calcination temperatures. All the samples display a type IV pattern with an H_3_ hysteresis loop, except for Co_3_O_4_/C−700 (black line). The Co_3_O_4_/C−750 exhibits the highest BET surface area of 139.8 m^2^·g^−1^, which is better than those of Co_3_O_4_/C−700 (42.3 m^2^·g^−1^), Co_3_O_4_/C−800 (80.6 m^2^·g^−1^), Co_3_O_4_/C−900 (47.4 m^2^·g^−1^) and Co_3_O_4_/C−1000 (44.9 m^2^·g^−1^). Combining the results of SEM, TEM images and TGA curve, we conclude that even though CoPc does not completely decompose at 750 °C, the resultant thin flake of the graphene-like carbon layer and evenly dispersed separate Co_3_O_4_ particles together lead to a higher specific surface area at this temperature.

### 3.2. Electrochemical Properties

Cyclic voltammetry (CV) and rotating disk electrode (RDE) measurements were applied to assess the ORR performances of the obtained catalysts. Figure 3a shows the CV curves of Co_3_O_4_/C−750 in O_2_−saturated (red line) and N_2_−saturated (black line) KOH solutions, respectively, implying that Co_3_O_4_/C−750 possesses obvious ORR activity. Figure 3b exhibits the ORR linear sweep voltammogram (LSV) curves of Co_3_O_4_/C−750 and 20% Pt/C. The half−wave potential (E_1/2_) of Co_3_O_4_/C−750 is 0.77 V, which is slightly lower than that of 20% for Pt/C (0.84 V). The RDE measurements with different rotating speeds (400–2000 rpm) were carried out (Figure 3c) to further assess the pathway of the ORR process over Co_3_O_4_/C−750, in which the limiting current density was enhanced with an increase in the rotation rate, indicating a shortened diffusion distance at higher rotation speeds [45]. Figure 3d displays the corresponding K−L plots from 0.3 to 0.7 V vs. RHE. The calculated result reveals that the number (n) of electrons transferred is ~3.7 for Co_3_O_4_/C−750, confirming that it is close to the four-electron ORR process on Co_3_O_4_/C−750 (details are shown in Supplementary Materials).

**Figure 4:**
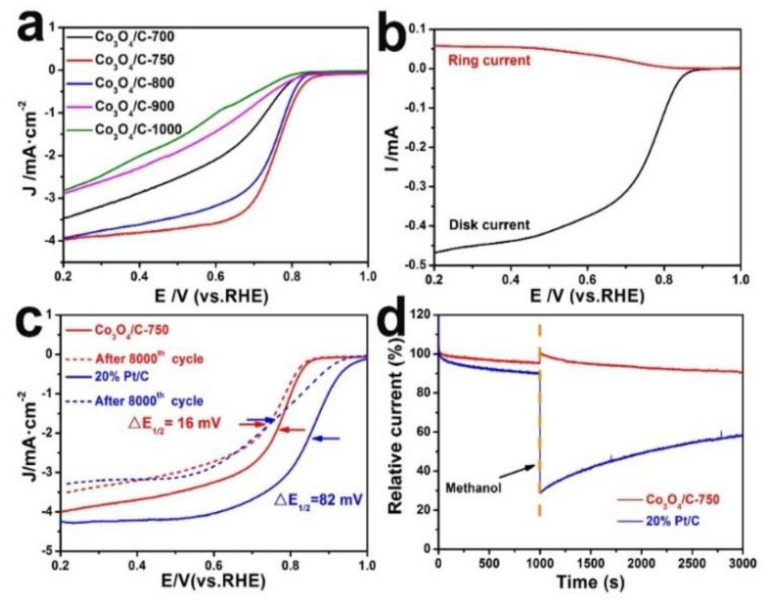
(**a**) LSV curves of Co_3_O_4_/C−700, Co_3_O_4_/C−750, Co_3_O_4_/C−800, Co_3_O_4_/C−900 and Co_3_O_4_/C−1000. (**b**) RRDE LSV curves for Co_3_O_4_/C−750, with GC disk−Pt ring electrodes at 1600 rpm in O_2_−saturated 0.1 M KOH solution (scan rate, 10 mV·s^−1^). (**c**) LSV curves of Co_3_O_4_/C−750 and 20% Pt/C after 8000 cycles in O_2_−saturated 0.1 M KOH solution. (**d**) LSV curves of Co_3_O_4_/C−750 and 20% Pt/C in 0.1 M KOH, with and without methanol.

Figure 4a shows LSVs curves of Co_3_O_4_/C−750 and other control samples, including the Co_3_O_4_/C−700, Co_3_O_4_/C−800, Co_3_O_4_/C−900 and Co_3_O_4_/C−1000 samples. As is shown, Co_3_O_4_/C−750 has relatively superior ORR properties, especially its half-wave potential (E_1/2_) of 0.77 V, outperforming those of the other samples. This can be attributed to the evenly dispersed separate Co_3_O_4_ particles and the graphene-like carbon layer structure, as well as its highest specific surface area among all the samples. The number of electrons transferred of Co_3_O_4_/C−750 was also computed to be 3.6 from the RRDE measurement (Figure 4b). The result is basically consistent with that derived from K−L plots (Figure 3d). It further proves that this is close to the four-electron pathway ORR process. Simultaneously, the electrochemical performances of pure Co_3_O_4_ and pure carbon black after 750 °C heat treatment were assessed, a procedure which was conducted (as shown in Figure S5) for the control experiments. The calculated numbers of electrons−transferred on the above two control samples (3.4 and 2.5, respectively) were significantly lower than those on the Co_3_O_4_/C−750 sample. Moreover, their half−wave potentials also could not reach 0.77 V. Besides a better ORR activity, Co_3_O_4_/C−750 also exhibits superior stability and good methanol tolerance. Figure 4c shows that after 8000 cycles, the half−wave potential (E_1/2_) of Co_3_O_4_/C−750 displays only a small left shift of ~16 mV, while 20% Pt/C gives substantial left shift of ~82 mV, confirming that Co_3_O_4_/C−750 has a better stability than 20% Pt/C. Figure 4d reveals the comparison of the durability between Co_3_O_4_/C−750 and 20% Pt/C via an evaluation of the chronoamperometric responses. A volume of 3 mL methanol was dropped into 97 mL 0.1 M KOH at 1000 s. The 20% Pt/C sample shows a significant decline, however, Co_3_O_4_/C−750 has no obvious decay, implying Co_3_O_4_/C−750 shows superior durability to 20% Pt/C. To further confirm the stability of Co_3_O_4_/C−750, the SEM/TEM images, XRD pattern and high−resolution XPS spectrum of Co 2p for Co_3_O_4_/C−750 after the ORR measurement were determined. As shown in Figure S6, there is almost no change on the morphology of the sample before and after the reaction. Figure S7a shows that the composition of Co^2+^ increased slightly after the reaction, indicating that a small part of Co^3+^ species were reduced to Co^2+^ species during the reaction. However, the XRD patterns of Co_3_O_4_/C−750 before and after the reaction exhibit negligible differences (Figure S7b). In sum, the above results fully demonstrate that Co_3_O_4_/C−750 possesses good stability.

**Figure 5:**
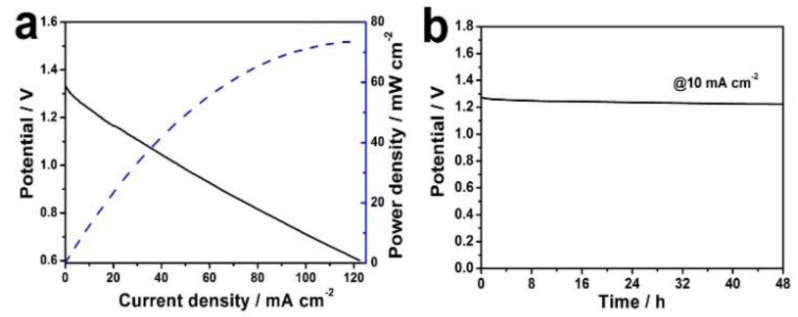
(**a**) Polarization and corresponding power density curves of Co_3_O_4_/C−750. (**b**) Galvanostatic discharge curve of Co_3_O_4_/C−750 at current density of 10 mA·cm^−2^.

In order to further explore the ORR properties of Co_3_O_4_/C−750, its application in a zinc–air battery was launched. The liquid mixture, dispersed with Co_3_O_4_/C−750, was dropped on a piece of carbon cloth as the cathode while a zinc sheet was used as the anode. Meanwhile, 6 M KOH was used as the electrolyte. Figure 5a presents polarization and corresponding power density curves of Co_3_O_4_/C−750. The open circuit voltage (OCV) of Co_3_O_4_/C−750 is 1.34 V and its power density can reach up to 74 mW·cm^−2^. In order to assess the stability of the battery, a galvanostatic discharge experiment of Co_3_O_4_/C−750 was performed. As shown in Figure 5b, there is no significant voltage reduction at a current density of 10 mA·cm^−2^ during the reaction time of 48 h, indicating that the zinc–air battery possesses good stability.

## 4. Conclusions

A series of Co_3_O_4_ supported on graphene-like carbon (Co_3_O_4_/C) compositeswere effectively synthesized by one−step calcination of CoPc. Based on the TGA curve of CoPc, the calcination temperatures were chosen as 700, 750, 800, 900 and 1000 °C. Among the obtained samples, the material pyrolyzed at 750 °C (Co_3_O_4_/C−750) shows the best ORR electrocatalytic performance (half−wave potentials of 0.77 V (vs. RHE) in 0.1 M KOH); moreover, it exhibits better stability and superior methanol tolerance than 20% Pt/C. The further electrochemical measurement results reveal that it is close in values to the four−electron ORR process on Co_3_O_4_/C−750. In addition, the application of Co_3_O_4_/C−750 in the zinc–air battery presents 1.34 V of open circuit potential. The enhanced electrocatalytic performance of Co_3_O_4_/C−750 can be ascribed to the synergistic effect between evenly dispersed separate Co_3_O_4_ particles and a thin−flake graphene−like carbon layer produced by pyrolysis of CoPc, as well as its high specific surface area. The obtained Co_3_O_4_/C−750 catalyst shows effective electrocatalytic activity under the condition of alkalinity, which could be considered as a promising nonprecious ORR catalyst for energy storage and conversion devices. Simultaneously, our study affords a feasible strategy for the development of effective electrocatalysts in zinc–air batteries.

## Data Availability

Data are available upon request from the corresponding author.

## Supplementary Materials

The following supporting information can be downloaded at: https://www.mdpi.com/article/10.3390/nano13071241/s1, Figure S1: (a) The structure diagram of CoPc. (b) TGA curve of CoPc under N_2_; Figure S2: SEM images of (a) Co_3_O_4_/C−700, (b) Co_3_O_4_/C−800, (c) Co_3_O_4_/C−900 and (d) Co_3_O_4_/C−1000; Figure S3: XRD patterns of Co_3_O_4_ (JCPDS No. 43−1003), Co_3_O_4_/C−700, Co_3_O_4_/C−800, Co_3_O_4_/C−900 and Co_3_O_4_/C−1000 (black, red, blue, pink and green lines, respectively); Figure S4: N_2_ adsorption–desorption isotherms of Co_3_O_4_/C−700, Co_3_O_4_/C−750, Co_3_O_4_/C−800, Co_3_O_4_/C−900 and Co_3_O_4_/C−1000 (black, red, blue, pink and green lines, respectively); Figure S5: (a) LSV curves for Co_3_O_4_ at different rotation speeds (400−2000 rpm). (b) K−L plots of Co_3_O_4_ at different electrode potentials (V vs. RHE). (c) LSV curves for carbon black after 750 °C heat treatment at different rotation speeds (400−2000 rpm). (d) K−L plots of carbon black after 750 °C heat treatment at different electrode potentials (V vs. RHE); Figure S6: (a) SEM image and (b) TEM image of Co_3_O_4_/C−750 after the ORR measurement; Figure S7: (a) High-resolution XPS spectra of Co 2p and (b) XRD patterns of Co_3_O_4_/C−750 before and after the ORR measurement. Section S1: Experimental section, structural characterization of CoPc and contrast samples, N_2_ adsorption–desorption isotherms of samples, and stability of sample after electrochemical test.
