# Supplementary material for: Co3O4 Supported on Graphene-like Carbon by One-Step Calcination of Cobalt Phthalocyanine for Efficient Oxygen Reduction Reaction under Alkaline Medium
Source: Nanomaterials (Basel). 2023 Mar 31;13(7):1241. doi: 10.3390/nano13071241 (PMC10097344; doi:10.3390/nano13071241)
Supplement: Supplementary file 1 [file nanomaterials-13-01241-s001.zip › nanomaterials-2293187-supplementary.pdf]

Supplementary Materials

Co_3O_4 Supported on Graphene-like Carbon by One-Step Calcination of Cobalt Phthalocyanine for Efficient Oxygen Reduction Reaction under Alkaline Medium

Section S1. Experimental section

1.1 Calculation of electron transfer number (n) for ORR

The Koutecky–Levich equation can be used for determining the electron transfer number (n):

$$\frac{1}{j} = \frac{1}{j_k} + \frac{1}{B\omega^{1/2}} \qquad (1)$$

$$B = 0.2nFC_0{D_0}^{2/3}\upsilon^{-1/6} \qquad (2)$$

Here, j is the measured current density, jk is the kinetic current density and ω is the rotating speed of electrode (rpm). B is determined from the slope of the Koutecky–Levich plots according to the Levich equation. n is electron transfer number per oxygen molecule, F is the Faraday constant (F = 96 485 C·mol^{-1}), C is the concentration of O_2 in the 0.1 M KOH solution (CO_2 = 1.2×10^{-6} mol·cm^{-3}), D is the diffusion coefficient of O_2 in the solution (DO_2 = 1.9×10^{-5} cm^2·s^{-1}), υ is the kinematic viscosity of the electrolyte (υ=0.01 cm^2·s^{-1}), and the constant 0.2 is adopted when the rotation speed is expressed at rpm. According to Equations (1) and (2), the number of electrons transferred (n) was calculated from the slopes of K–L plots to at potentials of 0.30 – 0.70 V. For RRDEs, the electron transfer number (n) was determined by the following equations, the disk electrode was scanned at a rate of 10 mV·s^{-1}, and the ring potential was constant at 1.6 V.

$$n = \frac{4I_D}{I_D + (I_R/N)} \qquad (3)$$

Here, ID, IR, and N are the disc current, the ring current, and the current collection efficiency of RRDE, respectively. In this equation, N = 0.43.

Supplementary Figures

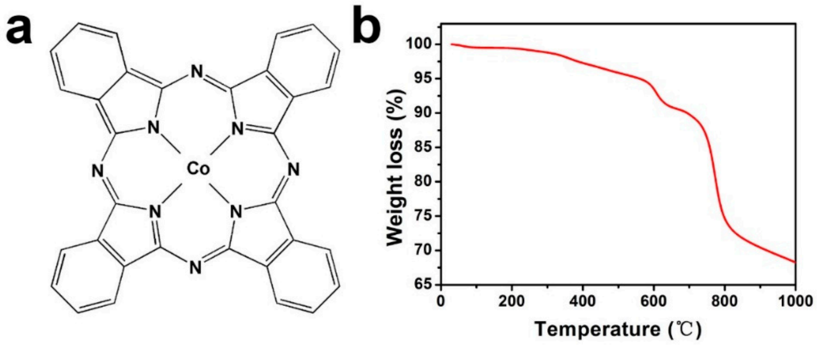

Figure S1. (a) The structure diagram of CoPc. (b) TGA curve of CoPc under N_2.

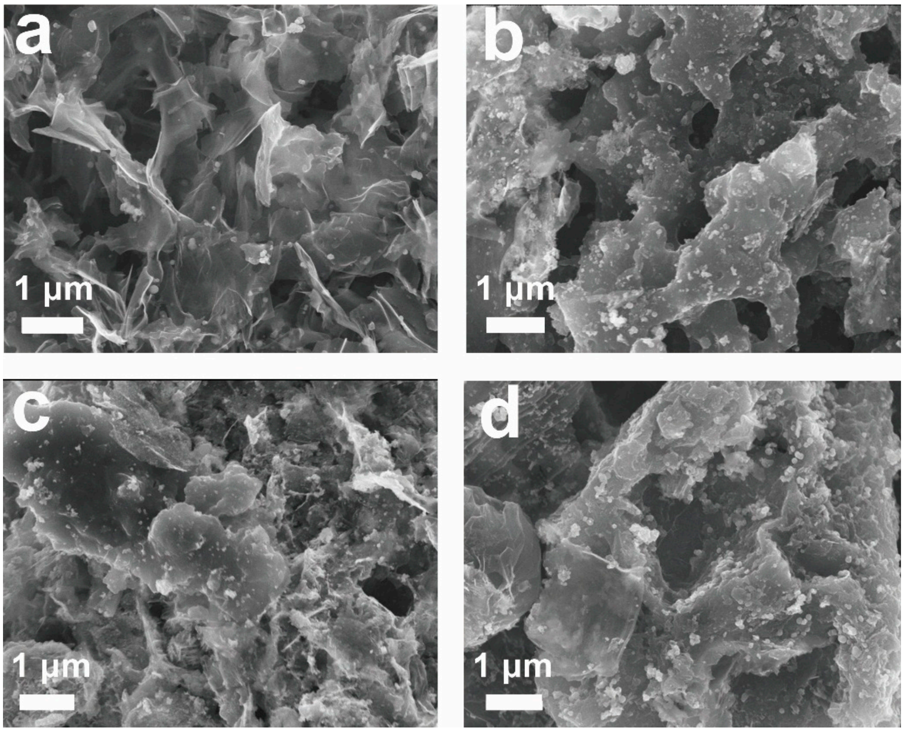

Figure S2. SEM images of (a) Co_3O_4/C–700, (b) Co_3O_4/C–800, (c) Co_3O_4/C–900 and (d) Co_3O_4/C–1000.

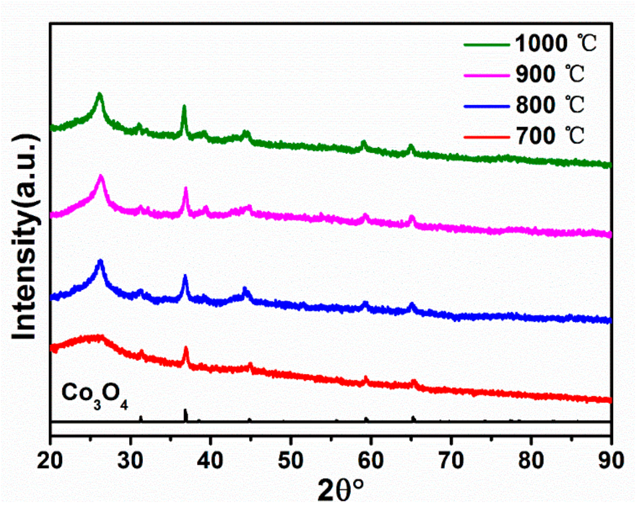

Figure S3. XRD patterns of Co_3O_4 (JCPDS No. 43–1003), Co_3O_4/C–700, Co_3O_4/C–800, Co_3O_4/C–900 and Co_3O_4/C–1000 (black, red, blue, pink and green lines, respectively).

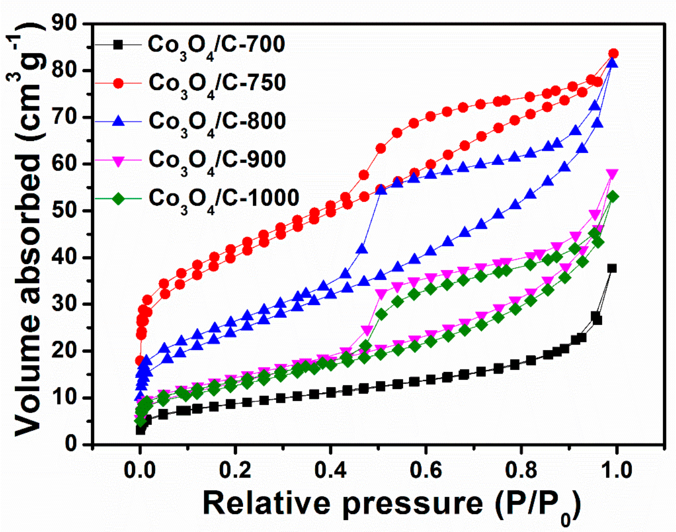

Figure S4. N_2 adsorptiondesorption isotherms of Co_3O_4/C–700, Co_3O_4/C–750, Co_3O_4/C–800, Co_3O_4/C–900 and Co_3O_4/C–1000 (black, red, blue, pink and green lines, respectively).

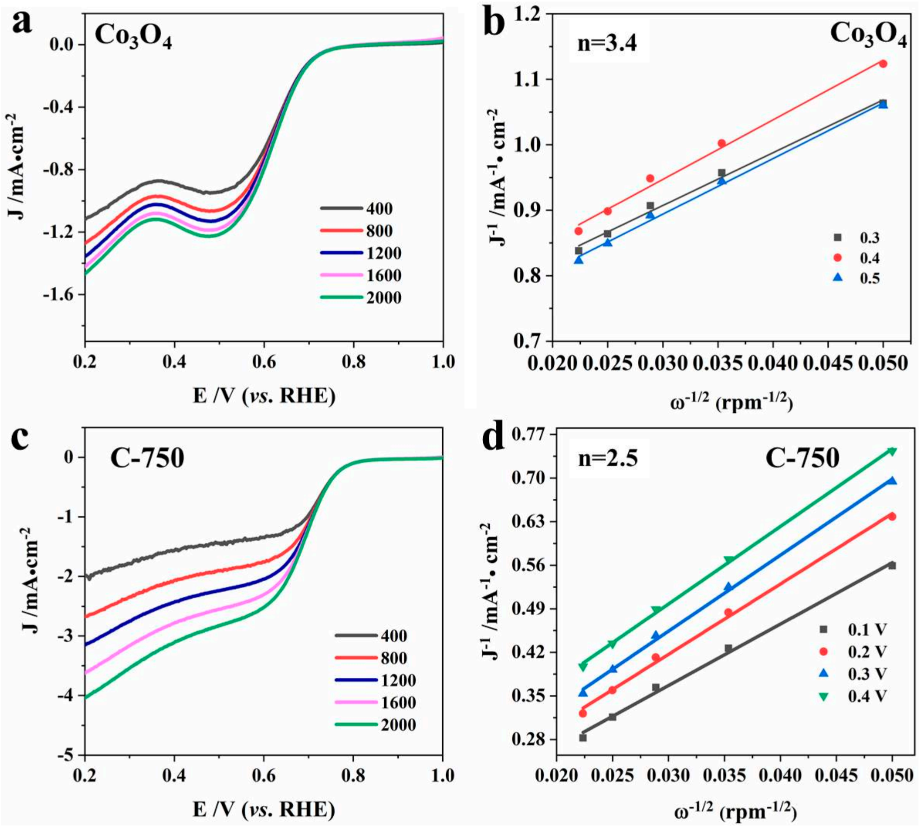

Figure S5. (a) LSV curves for Co_3O_4 (Sinopharm Chemical Reagent Co., Ltd) at different rotation speeds (400–2000 rpm). (b) K–L plots of Co_3O_4 at different electrode potentials (V vs. RHE). (c) LSV curves for carbon black (Sinopharm Chemical Reagent Co., Ltd) after 750 ºC heat treatment at different rotation speeds (400–2000 rpm). (d) K–L plots of carbon black after 750 ºC heat treatment at different electrode potentials (V vs. RHE).

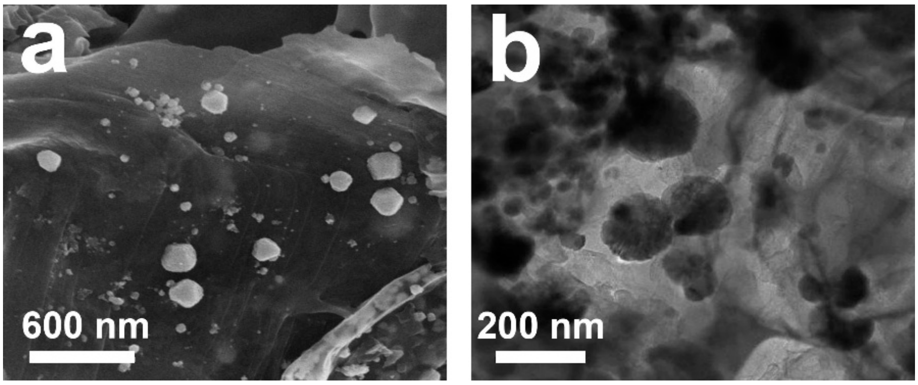

Figure S6. (a) SEM image and (b) TEM image of Co_3O_4/C–750 after the ORR measurement.

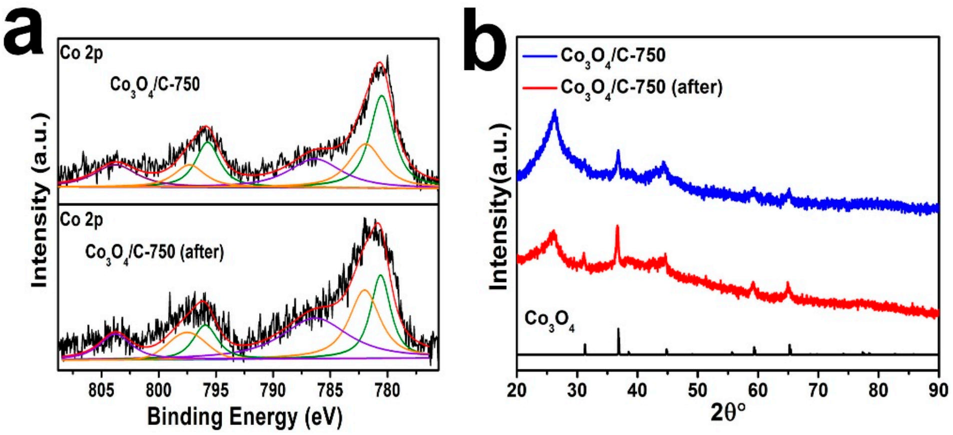

Figure S7. （a) High–resolution XPS spectra of Co 2p and (b) XRD patterns of Co_3O_4/C–750 before and after the ORR measurement.
